# New insights into crisis communication from an “inside” *emic* perspective during COVID-19

**DOI:** 10.1177/2046147X21999972

**Published:** 2021-05

**Authors:** Jim Macnamara

**Affiliations:** University of Technology Sydney, Australia

**Keywords:** COVID-19, crisis communication, crisis response, emic research, reputation

## Abstract

As the COVID-19 pandemic spread across the world, requiring emergency management by health authorities and providers, it created flow-on crises and “crisis contagion” for organizations ranging from international airlines and tourism operators to local businesses, schools, and universities. In addition to the risks directly associated with the health emergency, many organizations were plunged into crisis because of severe restrictions to their operations and income losses. This analysis examines crisis communication in an organization faced with major financial losses, staff redundancies, and disruption. It analyses how these and necessary crisis responses were communicated to stakeholders, using *situational crisis communication theory* (SCCT), as its analytical framework. While noting alternative perspectives such as *crisis and emergency risk communication* (CERC) theory, SCCT is identified as the most widely applied theory of crisis communication, and thus warrants ongoing review in an era of media fragmentation, disinformation, and low public trust. Furthermore, this analysis provides a relatively rare “inside” (*emic*) perspective through ethnography and autoethnography conducted by a senior decision-maker in the organization studied, which expands traditional outside (*etic*) perspectives and offers new insights into crisis communication.

## The unprecedented COVID-19 crisis—and its related crises

COVID-19, a highly infectious coronavirus,^1^ caused the largest global crisis in terms of human health risk, impact on economies, and social and political upheaval since the Second World War. This particular strain of coronavirus, which was first reported in Wuhan City in Hubei Province, China in December 2019 (thus named COVID-19), spread rapidly to more than 100 countries by March 2020 and was declared a pandemic by the World Health Organization (WHO) on 11 March 2020 (World Health Organization, 2020a).

As widely reported, the pandemic led to an almost complete shutdown of all but essential services and businesses in hundreds of cities; self-isolation by millions of people worldwide; and near overwhelming demands on health systems as hundreds of thousands of people became seriously ill, and many died. As 2020 drew to a close, infections were approaching 100 million and deaths neared 2 million worldwide (Worldometer, 2020).

The virus, and attempts to contain it, in turn resulted in the loss of more than 1 billion jobs temporarily or permanently (International Labour Organization, 2020), as businesses and service providers closed down, leading to widespread economic hardship and escalating anxiety and even panic among populations. Compared with crises analyzed in academic literature over several decades, the impact of the COVID-19 pandemic on organizations in many sectors was unprecedented and beyond the scale of most crisis management and crisis communication case studies.

COVID-19 undoubtedly will be the subject of numerous studies from an emergency management and health perspective. The focus of this analysis is not emergency management of the pandemic or health communication, but the communication challenges of an organization in coping with major disruption to and adjustment of its operations caused by emergency response strategies such as travel restrictions and “lockdowns” of workplaces and various services.

The spread of COVID-19 resulted in “crisis contagion” as well as viral contagion, as its effects “spilled over from one organization to another” (Laufer and Wang, 2018: 173) and resulted in events and circumstances that created major negative impacts and challenges for many organizations. While initially and centrally a health emergency, the pandemic rapidly spread causing a range of “knock on” impacts. What is analyzed here is organizational management and communication related to the chronic operational, financial, and potential reputational crisis faced by a large public sector organization caught in crisis contagion (see 2.1, “Understanding what constitutes as crisis”).

In this instance, the organization studied is in Australia, a country that responded early and strongly to the declaration of the pandemic. Australia banned international travel except in special circumstances such as repatriation of citizens; organizations other than essential services were ordered to direct staff to work from home; and complete lockdowns were implemented in “hot spots” of community transmission, including entire cities in some cases. Such emergency management strategies were seen as necessary and they restricted COVID-19 infections in Australia to 28,408 and deaths to just over 900 as at the end of 2020 (Department of Health, 2020). However, there were potentially catastrophic financial and operational impacts and reputational damage for organizations if they did not implement effective crisis management and crisis communication, as will be explained.

A feature of this analysis is that it is based on an *emic* autoethnographic and ethnographic approach that accesses an “inside” perspective from a participant directly involved in decision-making and communication. This afforded a “close up” perspective (Heide et al., 2018: 454), in contrast with the vast majority of studies of crisis management and crisis communication that are undertaken from an outside observer or independent analyst (*etic*) standpoint. The benefits of this approach are discussed under “Methodology” and in the findings.

## The theory of crisis management and crisis communication

### Understanding what constitutes a crisis

In the foreword to the *Handbook of Crisis Communication*, Heath (2010: 3) notes that around 20 definitions of a crisis have emerged in public relations and corporate communication literature. In a chapter of the handbook, Coombs lists eight of these, but in discussing “key definitions of a crisis,” he settles on his own 2007 definition in which he described a crisis as “an unpredictable event that threatens important expectations of stakeholders and can seriously impact an organization’s performance and generate negative outcomes” (Coombs, 2007a: 2–3). Elsewhere, Coombs describes a crisis as “a significant threat to operations that can have negative consequences if not handled properly” (Coombs, 2007b: para. 2, 2014b: para. 3). Coombs (2014a, 2014b) does note that, as well as causing financial and reputational damage for organizations, crises can cause public harm such as injuries or loss of lives (para. 3). However, evident in these definitions and in most public relations and corporate communication literature is a focus on crises as an *event*, or *event related*, and on the effects of crises on organizations and communication to protect the interests of organizations. In an extensive review of crisis management and communication literature, Kent (2010) observed that “nearly every conference paper and article implicitly or explicitly treats crisis from the standpoint of the organization” (p. 705).

Political scientists and emergency and disaster management specialists define a crisis more broadly as “a phase of disorder in the development of a person, an organization, a community, an ecosystem, a business sector, or a polity” (Boin et al., 2017: 5). Sociological studies also examine crises through a wider lens, focusing on risks to individuals, groups, communities, or social systems and society as a whole. Anthropological literature, in particular, presents a different perspective to Coombs’ association of a crisis with an event, defining a crisis as “conditions” in which “people (including the ‘state’s agents’) must cope with a variety of unexpected disruptions” (Greenhouse, 2002: 8). These broader concepts of crisis are useful in examining the range of disruptions and conditions with which people and organizations had to cope following the outbreak of COVID-19.

### Types of crises

Lerbinger (1997) identified eight types of crisis as (1) natural disaster; (2) technological crisis; (3) confrontation; (4) malevolence; (5) organizational misdeeds; (6) workplace violence; (7) rumors; and (8) terrorist attacks and other “man-made” disasters.

In his extensive writing on crisis management and communication, Coombs (2007a, 2007c, 2015, 2018) has identified a similar range of crisis events and categorized them into three types based on the likely attribution of blame and public expectations in relation to response, which he terms *crisis responsibility*.

While Lerbinger (2012) later grouped crisis events into three categories, which he labeled (1) *physical* crises caused by external factors beyond the control of the organization such as natural disasters and possibly including technological or biological crises; (2) *human-climate* crises created by an external stakeholder acting in conflict or malevolently; and (3) *management failures*, the events and effects examined in this analysis do not neatly fit into any of the above categories. The existential threat posed by a pandemic and its flow-on effects were not a “natural disaster” by most definitions,^2^ and the range of impacts were not “man-made.” In recent writing, Coombs (2019) advocates a broader understanding of crises, but he continues to focus on organizational perspectives and interests.

Coombs (2015) categorizes crises as (1) *victim* crises such as natural disasters, damaging false rumors, and product tampering by an external actor; (2) *accidental* crises such as industrial accidents beyond an organization’s control; and (3) *intentional* crises involving organizational misdeeds such as fraud, safety breaches, or negligence (p. 264). He referred to the third category as *preventable* crises in his earlier work (Coombs, 2007c: 168). Based on *attribution* theory (Heider, 1958), Coombs (2007c: 168, 2015: 264) goes on to argue that victim crises result in “very low attributions of crisis responsibility” and that accidental crises result in “minimal attributions of crisis responsibility,” with only the intentional crisis cluster leading to “strong attributions of crisis responsibility” and “severe reputational threat.”

### Crisis communication

The predominant body of theory in relation to crisis communication in public relations and corporate communication is situational crisis communication theory (SCCT) (Coombs, 2007c, 2015, 2018). Central to SCCT is identification of *crisis response strategies* for the three types of crisis identified by Coombs—natural disasters, accidental crises, and intentional crises. Coombs proposes four key crisis response strategies with 10 specific executions or sub-types, as shown in Table 1.

**Table 1.** Crisis response strategies proposed by Coombs (2015: 266).

Response strategy	Sub-types	Management action
Denial	Denial	Claim that no crisis occurred
	Attack accuser	Confront the person or group claiming a crisis
	Scapegoat	Blame some outside person or group
Diminish	Excuse	Deny intent to do harm/claim inability to control events
	Justification	Minimize the perceived damage caused
Rebuild	Compensation	Offer money or other gifts to victims
	Apology	Accept responsibility and ask stakeholders to forgive
Bolstering	Reminder	Tell stakeholders about past good works
	Ingratiation	Thank or praise stakeholders for their help
	Victimage	Remind stakeholders that the organization is also a victim

The crisis response strategies proposed by Coombs are determined based on *crisis responsibility* and focused on avoidance of blame and resulting reputational damage. In this regard, SCCT notes that various *intensifiers* can exacerbate the level of responsibility, and therefore blame, attached to organizations. Intensifiers include the organization’s history of crises, its performance history (i.e. track record), and sometimes the severity of damage caused. A positive performance history and reputation does not bestow a “halo effect” as often claimed, according to research. However, the converse—a poor track record and reputation—can result in a “Velcro effect,” according to Coombs (2015: 265). He argues that the presence of negative intensifiers such as a previous history of crises and/or poor performance can result in a victim crisis being viewed as an accidental crisis and an accidental crisis being viewed as an intentional crisis (Coombs, 2015: 265).

A further key feature of SCCT is that media coverage is seen as having a critical role both in shaping the reputation of an organization before and during a crisis and as a channel of communication during and after a crisis (Coombs, 2015: 271). Drawing on turn-of-the-millennium research by Deephouse (2000), Coombs (2015) says that “most of the information stakeholders learn about a corporation is derived from media reports” (p. 271). Many other public relations researchers also pay considerable attention to media relations and media publicity as part of crisis management and crisis communication (e.g. Fearn-Banks, 2007; Verhoeven et al., 2014). Coombs (2007c) does refer to the internet and intranets, but he has consistently highlighted news media as playing a key role (p. 164) and, in recent revisions of SCCT, has focussed on the role and impact of social media (Coombs, 2014b, 2018).

Crisis communication theory is also influenced by studies of *image restoration* or repair (Benoit, 1995; Coombs, 2006), *impression management* (Coombs, 2006; Schwarz, 2008: 32), and *apologia* (Coombs, 2006, 2015; Frandsen and Johansen, 2010; Schwarz, 2008: 32)—a point noted by Arendt et al. (2017) in their study of more than 100 peer-reviewed articles on crisis communication. Coombs (2015) acknowledges that traditionally “study of crisis response strategies has been heavily skewed toward apologies” (p. 273). As well as illustrating a focus in public relations literature on the organization (e.g. its image), these theories further illustrate a focus on fault and defending against attribution of blame, or what Coombs (2015) calls “crisis responsibility” (p. 267).

There are also a number of functions and practices that are closely related to and sometimes overlap with crisis communication as it is theorized in public relations and corporate communication literature. These are briefly noted as context and as part of the analytical framework where they are applicable.

### Crisis management

Crisis management and crisis communication are sometimes conflated and treated as synonymous. For example, public relations literature has often claimed that the function is responsible for crisis management (Fall, 2004). This is contestable and was incorrect in the case examined here, as will be explained. Nevertheless, the practices of crisis management and crisis communication are closely integrated.

Coombs (2014a) says “crisis management is a process designed to prevent or lessen the damage a crisis can inflict on an organization and its stakeholders” and he identifies three phases in crisis management as pre-crisis, crisis response, and post-crisis (para. 5). Significantly, this definition of crisis management extends recognition of those affected beyond the organization to *stakeholders*, as does that of Bundy et al. (2017), which defines crisis management as “the process by which an organization deals with a disruptive and unexpected event that threatens to harm the organization or its stakeholders” (p. 1661). Boin et al. (2017) also observe that crisis management involves “a set of interrelated and extraordinary governance challenges” (p. 4).

### Crisis leadership

Along with a number of leadership and management scholars, Boin et al. (2017) also highlight the importance of leadership in a crisis. In *The Politics of Crisis Management: Public Leadership Under Pressure* they say: “in . . . times of crisis, citizens look to their leaders . . . leaders are expected to chart pathways out of the crisis” (p. 3). Relevant to this analysis, they say: “The job of crisis leadership . . . is to limit the depth and duration of the chaos, bewilderment, helplessness, and anger that this tends to cause, and to mobilize and harness coping capacity from within the community” (p. 12).

There is much focus on crisis “management” in crisis literature. But leadership is identified as different to management in several key respects, such as having an outward versus inward focus and a focus on change and looking to the future rather than stability and preserving or re-establishing the status quo, which is a key focus of management (Lunenberg, 2011). It can be argued that greater attention to leadership in a crisis is important. A first-hand perspective of leadership, as discussed here, offers a contribution to such study.

### Emergency management

Emergency management is related to crisis management to some extent, and some use the terms interchangeably. However, Boin et al. (2017), Haddow et al. (2017, Reynolds and Seeger (2005), and others identify emergency management with risk assessment, preparedness, and management of natural and technological hazards that require the intervention of emergency services, such as hurricanes, tornadoes, tsunamis, floods, wildfires, earthquakes, volcanic eruptions, dam failures, nuclear accidents, hazardous materials incidents, and terrorism, as well as disease outbreaks and pandemics. As this analysis is focussed on an organizational crisis triggered by emergency management measures, rather than management of the emergency, crisis management and crisis communication theory provide a more relevant analytical framework.

### Emergency and risk communication

While Coombs (2014a) identifies pre-crisis communication as one of three stages, *risk communication* focusses on communication for minimizing risks and avoiding emergencies and disasters, such as through warnings and public education. Covello (1992: 359) defines risk communication as “the exchange of information among interested parties about the nature, magnitude, significance, or control of a risk.” Renn (2008) and others draw attention to the multiple dimensions and objectives, as well as the context, of risk and emergency communication, which can include persuasion (e.g. health advice), as well as responding to *information seeking*.

Recently, Reynolds and Seeger argued that it is productive to combine crisis communication and risk communication. While noting differences, Reynolds and Seeger (2005) say that risk and crisis communication “have much in common and intersect at a variety of points” (p. 47). They note that public relations approaches to crisis communication are primarily focussed on *crisis events* and that they are mainly concerned with protecting the reputation of the organization. They propose instead a five-stage approach, which they call *crisis and emergency risk communication* (CERC), that takes a broader view of crises from pre-event stages to “eruption” and then “post-mortem and clean up stages” and addresses risks to society as well as key stakeholders. As noted, crisis communication literature addresses pre-event preparedness and post-crisis actions to some extent. However, given the focus of Reynolds and Seeger (2005) on developing a “comprehensive approach to emergency public health events” (p. 44), and their specific reference to infectious disease outbreaks and epidemics as examples of a “chronic crisis” that develops over longer periods of time than traditional event crises (p. 51), CERC is also relevant to this analysis.

## Methodology: A “close up” insider’s perspective

This analysis stems from a situation in which the author, an academic researcher, was also a senior executive in a large Australian public sector organization responsible for leading and managing its operations during and following to the outbreak of COVID-19. The organization is a faculty of a university ranked in the “top 150” in the world in 2020 by one of the leading ranking systems (Quacquarelli Symonds, 2020). The faculty, made up of two schools and eight research institutes and centers, had more than 4000 students including more than 400 higher research degree students and almost 400 academic, administrative, and technical staff at the time. Thus, it represents an enterprise of significant size. Furthermore, the operations of the faculty are important in this context, being the Faculty of Science of the university specializing in life sciences including biology, medical microbiology, metabolic biochemistry, parasitology, and research to combat infectious diseases, as well as mathematical and computer sciences. Upon the outbreak of COVID-19, the faculty was required to continue a number of its operations not only to support students, but to maintain vital research into vaccines, testing, and immunology being undertaken in collaboration with universities and research institutes worldwide. Also, the faculty held bacterial and viral cultures, animals, and cadavers in its laboratories, which needed to be maintained in a hygienic and safe way. These factors presented a series of significant operational challenges as well as major risks in terms of physical health and safety, mental health and wellbeing, animal welfare, and financial sustainability.

### The site of research

While having a disciplinary background in media and communication, the author was contracted as Deputy Dean of the Faculty of Science of the university prior to and during the outbreak of COVID-19, and was Acting Dean during the first critical weeks of the crisis in early 2020. Key response strategies were developed during this period, including the cancellation of all on-campus classes and moving more than 300 academic subjects and 4000 students to online learning; transitioning almost 90% of staff to remote working; and managing sensitive issues such as the termination of many short-term and casual staff contracts. Also, several students tested positive to the coronavirus during the transition period, requiring urgent tracing of close contacts, isolation, cleaning, and communication in an environment of high anxiety and fear.

After the initial peak period of activity following proclamation of the pandemic in early March 2020, the researcher continued as Deputy Dean and a member of the faculty executive team (FET)^3^ responsible, or jointly responsible, for high-level crisis management and communication throughout 2020.

The Deputy Dean role also included membership of the university’s senior leaders’ group (SLG) and a number of university committees, involving leadership responsibilities and direct access to and participation in decision-making affecting the university’s 3500 staff and 45,000 students in total. Thus, this analysis constitutes a “close-up” study, which Heide et al. (2018: 454) identify as rare and needed to provide “richer and more varied empirical material” in relation to strategic communication.

### An emic (insider) versus outside (etic) perspective

As noted in the introduction, this analysis offers a different and important contribution to literature in the field from an *emic* (inside) perspective. While offering independent critique, the authors of crisis-related studies rarely if ever are a member of the senior executive team responsible for an organization’s key decisions and its *stance*. Even when communication practitioners are part of the crisis management team (CMT) and give strategic advice as well as distribute information, the stance of an organization is determined by others. The stance of an organization is recognized in *contingency theory* as a fundamental determinant of what it does and says (i.e. strategy), and of stakeholder and public response (Cameron et al., 2008). In the case of crisis communication, Coombs (2014a) notes that an organization’s stance “denotes how it responds to conflicts” (p. 18). Thus, there is much to be learned from the first-hand view of an “insider” directly involved in making key decisions and statements in a crisis.

### Approach

This emic analysis is based on a combination of ethnography and autoethnography undertaken within an interpretivist humanistic approach. As L’Etang et al. (2012) noted in an editorial in a special issue of *Public Relations Review*, “anthropology, and its methodological approach—ethnography—seem natural bedfellows for public relations scholarship and practice” (p. 519), and the special issue advocated increased ethnographic as well as autoethnographic research. Waymer and Logan (2016) argue that autoethnography “has the potential to shed unique insights into the lived work experience of key organizational stakeholders” (p. 1458).

As well as involving self-reflection on the author’s own experiences and actions, the discussion and conclusions are based on first-hand observation of decisions and actions taken by the various leadership and executive committees of the faculty and university during the series of major disruptions and unprecedented conditions that followed the outbreak of COVID-19.

While some are critical of ethnography, and particularly autoethnography, on the grounds of subjectivity, “self-representation” as discussed in the following, and a lack of empirical evidence in some cases (Denzin and Lincoln, 2008), a body of knowledge has accumulated to inform observation and self-reflection as valid qualitative research methods.

Creswell (2009) defines ethnography is “a strategy of inquiry in which the researcher studies an intact cultural group in a natural setting over a prolonged period of time by collecting, primarily, observational and interview data” (p. 13). In addition to identifying *observation* as a primary research method used in ethnography, Geertz (1973) cited *participation* by the researcher in the activities studied as a valid contribution to data collection and reflective analysis. The result of first-hand observation and participation over a period of time results in what Geertz calls “thick description”—that is, a deeper level of understanding and analysis than that afforded by social science research methods such as self-reporting surveys and single-shot interviews. Elaborating on Geertz’s description, Neuman (2006) emphasizes that ethnography is “very detailed description of a . . . culture from the viewpoint of an *insider* in the culture to facilitate understanding of it” (p. 381) [original emphasis].

In a review of authoethnography in an international encyclopedia of communication research, Adams et al. (2017) similarly note that autoethnographers can “articulate insider knowledge of cultural experience” (p. 3) and also that “authoethnographers can write about experiences that happen in private” (p. 4), which are invisible to outside researchers, and they can describe “how being there in the experience *feels*,” not simply report phenomena and facts (p. 3) [emphasis added]. In their call for attention to autoethnography, Waymer and Logan (2016) draw on Bullough and Pinnegar (2001) to point out that the methodology can “articulate nodal moments”; “provide fresh perspectives on established truths”; and identify “complication or tension” (p. 1463).

Autoethnographers vary in their emphasis on *auto* (self), *ethno* (sociocultural connection), and *graphy* (the application of research methods and processes) (Wall, 2008: 39). Researchers using autoethnography are urged to draw on empirical data as far as possible, rather than relying solely or largely on personal observations that can result in memoir or ego-driven representation to project their “self-worth” (Tedlock, 2000: 468). The “self” in autoethnography serves as the vantage point, which affords direct access to data that otherwise might not be available. What separates ethnographic and autoethnographic research from autobiographical stories and memoirs, according to Maréchal (2010), is reflexivity and the connection of observations to wider cultural, political, and social understandings. This connection involves supporting personal observation with records, correspondence, and other evidence, as noted in the following.

### Methods

In addition to direct personal observation, ethnography and autoethnography are operationalized through the collection and analysis of documents such as journal notes and diaries. In an organizational context, records that support observation also include minutes of meetings, e-mails, reports, website and intranet statistics,^4^ and research such as surveys. During this research, a detailed diary was kept, along with extensive journal notes, and a range of the above organizational data sources were accessed, as reported under “Observations, reflections, and discussion.”

As noted by a number of qualitative researchers, observation in ethnography and autoethnography is also supported by interviews with key informants in some cases. In this case, formal interviews were not conducted because the researcher was in daily conversations and e-mail communication with all members of the FET and the university’s senior management, as well as staff and students. When “social distancing”^5^ was introduced, regular meetings were conducted via Zoom and Microsoft Teams. All online meetings and presentations were recorded and the Governance Manager of the faculty maintained an archive of all announcements, guidelines, and responses issued by the leadership team, affording a comprehensive record of decisions, actions and communications, as well as feedback from key stakeholders. The research proceeded inductively, as is common in ethnography and autoethnography, rather than in response to a series of research questions. The research sought in an open-ended way to identify (1) the major challenges in dealing with the crisis; (2) the key strategic decisions and actions taken including their rationale; (3) the response and outcomes of those decisions and actions as far as could be determined; and (4) to compare these with crisis management and crisis communication theory.

While this analysis is based on less than 2 years study, which Tedlock (2008: 151) recommends as ideal for ethnography, the 9 months from March to December 2020 was an intense period in terms of management and communication in relation to changes and actions necessitated by COVID-19. Also, this analysis arguably provides “thick description” because of the proximity of the researcher to “the action.”

## Observations, reflections, and discussion

The following analysis, based on observation, reflection and records, reviews key leadership, management, and communication initiatives in the context of the conceptualization and theorization of crisis management and crisis communication in the literature, with particular attention to communication.

To understand the context of management decisions and communication, it is necessary to note that the national government closed the nation’s borders to all but returning citizens within days of declaration of COVID-19 as a pandemic. This meant that more than 10,000 international students enrolled at the university could not enter the country to commence or continue study in 2020, including more than 1000 studying in the Faculty of Science. This resulted in a loss of more than $200 million of annual income to the university and a $12–$15 million annual loss to the faculty. Also, a range of health authority regulations and advisories were issued requiring physical distancing between people and restrictions on the size of gatherings. While universities were classified as essential services and exempted from some restrictions, social responsibility as well as legal and operational imperatives guided management decisions.

A single paper cannot discuss the extensive range of decisions and actions taken during the crisis. However, for broad context, some of the major operational initiatives implemented by the faculty in conjunction with university management, which had to be communicated to stakeholders, are summarized in Table 2.

**Table 2.** Key organization initiatives and issues that required communication.

Stakeholder	Initiative	Key decisions and actions
Employees	Social distancing	• 1.5 m physical distancing policy implemented including in laboratories (see “Researcher safety”) • All non-essential travel cancelled • Self-isolation by any staff with symptoms, in accordance with government guidelines
	Working from home	• Directed staff to work from home unless involved in essential work (e.g. vaccine research; security) • Health and wellbeing advice for remote working provided on the staff intranet • IT infrastructure upgraded including expanded Zoom capacity and internet bandwidth
	Researcher safety	• Recalled all researchers working overseas • Deferred projects when possible, particularly close contact laboratory work • Additional personal protection equipment (PPE) provided in labs (i.e. gowns, masks, and gloves)
	Laboratory safety	• Delivery of cadavers to anatomy labs suspended • Emergency team established to protect cultures, animals and chemicals in labs
	Casual staff reduction	• Casual staff contracts reduced by 30% due to reduction in weekly classes through conversion to online learning • Expiring short-term contracts not renewed • Casual staff “hardship fund” established
Students	Remote/online learning	• 300 subjects converted to online learning in 4 weeks. Included “low-tech” solutions for students behind restrictive firewalls in China
	International students	• Maintained contact with international students offshore, particularly in China • Offered online learning (as above)
	Local students	• Subject coordinators contacted all local students by e-mail with regular updates
	Students tested positive	• Telephone contact with students tested positive • Telephone contact with teaching staff in contact • E-mail communication to all students in contact based on class lists
Financial	Expenditure reductions	• Recruitment freeze implemented immediately • 90% of planned capital expenditure postponed • Voluntary separation package (VSP) offered mid-year. Reduced university staff by 357; reduced Science staff by 24 • Additional 100–150 redundancies announced to meet reduced 2021 budget • Identified some assets for sale to generate cash
Research funders	Research grants and contracts continuity (*e.g. renegotiate deadlines*)	• Research Office communicated directly with national research grants bodies • Lead researchers communicated with contract research funders • Engagement Office communicated with philanthropic donors
Government	Policy and regulation compliance	• University liaised daily with national and state government, including the Department of Health • Offered staff and laboratories to help in COVID-19 testing and production of hand sanitizer

In relation to these and other decisions taken, the overwhelming sense derived from the period leading the faculty during the early days of the COVID-19 pandemic was the unrelenting demand for numerous decisions to be made and explained under intensive time pressure, and the near insatiable demand for information, in an environment of rapid change. Information was being received from the federal government, the state department of health, local health authorities, international authorities such as the WHO, and the university’s central administration. Sometimes there were contradictions, and often there were gaps in information. Getting things right was an ideal in direct conflict with pressures to act. Going slow was not an option. In this crisis, people could die, or in the least become seriously ill, if decisions were not made and information was not provided in a timely manner.

Leadership and management also required careful balancing of interests. For example, decisions such as whether to shut a laboratory or clinic, or cancel classes, had to be cognizant of the differing impact on multiple stakeholders. With some laboratories involved in infectious disease research and others transitioning their efforts to large-scale COVID-19 testing based on next-generation DNA sequencing and bulk production of hand sanitizer, a full shutdown as recommended by some was not necessarily the best decision for society. Transparency had to be balanced with privacy in cases of staff or students reporting contact with someone who tested positive to COVID-19. Pragmatism, such as terminating casual contracts related to cancelled classes, had to be balanced with consideration of human welfare and workforce loyalty, knowing that many of the same people would be needed once the crisis had passed. Phrases such as “a tough day at the office” were recorded several times in the author’s journal, along with the confessional statement “We hurt a lot of people today,” referring to the tension between sustainable operational management and human welfare.

### Learnings for crisis leadership, management, and communication

Reflection and discussion with faculty executive colleagues in FET meetings (an example of accessing private information through ethnography and autoethnography) afforded or confirmed a number of learnings for leadership and senior management, including the following.

(i) Leaders and senior managers have to be *agile* and capable of processing and weighing up large amounts of information from multiple sources to make decisions. For example, advisories from health authorities were frequently updated, sometimes several times a day, requiring adjustment to operations and regularly updated communication with stakeholders.
(ii) Accessing and processing large amounts of information required systematic and multi-level *monitoring*. While traditional and social media monitoring, which is emphasized in much crisis communication literature, revealed public crisis narratives including disinformation and politicized discourses, it was necessary to closely monitor the websites of relevant authorities to access official information and to monitor e-mail that reflected the questions and concerns of employees and other key stakeholders.
(iii) Leaders and senior managers need to be *visible and engaged*, particularly with employees and closely connected stakeholders such as customers (students in this case). Despite the health risks, staff and students of the faculty expected to see and hear from their leaders on a regular basis. This aligns with international studies that show “leading by example” as the public’s highest expectation of leaders (Moreno et al., 2018: 94). As Deputy Dean, and Acting Dean in a key period, the author was present in the faculty office from 7 am on week days and available on video conferencing applications such as Zoom and a cell phone 24/7. Even when physical distancing and working from home were implemented, the faculty executive team maintained a roster of senior executives on site given that some operations continued because the faculty was declared part of an essential service. “Walk arounds” to laboratories and occupied offices undertaken by the author and other members of the FET were greeted with enthusiasm and gratitude by staff members, with frequent comments such as “Thank you for coming to see us” and “It’s good to see someone from management.”
(iv) Crisis management teams (CMTs) need to be small and centrally located, as large and dispersed management teams become difficult or impossible to coordinate and lose consistency of messaging, leading to conflicting advice and chaos. The *composition of the CMT* and its operation are discussed further in a following section.
(v) Leaders and senior managers need to *speak directly, frankly*, and *authentically* to stakeholders—not through statements written by human resources (HR), marketing departments, or through media releases. “Communicating in an open and transparent way” has been identified as the second most cited public expectation of leaders after “leading by example” (Moreno et al., 2018: 94). Implications of this for public relations, marketing, and other functional units are discussed further in the following.

On 30 March 2020, just two and a half weeks after declaration of the pandemic, a long-serving senior academic staff member in one of the faculty’s research centers committed suicide at home. While the staff member had a history of mental health problems, the news caused shock and grief among colleagues, as could be expected. It also was personally upsetting and confronting for the faculty’s senior executives including the author. But urgent empathetic communication was required with staff in the research center, as well as the staff member’s family, so personal emotional responses had to be put to the side. Fortunately, the faculty’s leaders had support from psychologists in the university’s HR department. However, stress and the effects of stress are factors to be considered in crisis communication. In addition to this very upsetting incident, other examples of stress and their effects are reported later in both personal reflections and comments from faculty executives (see Section 4.8).

A number of specific findings that inform crisis management and crisis communication theory and practice emerged from first-hand “lived experience” followed by reflection and review of documents and records. The most noteworthy findings are reported in the following, together with additional personal reflections and insights.

### Crisis type expansion

First, none of the crisis types identified in SCCT were directly relevant for the university faculty during the COVID-19 pandemic. The flow-on effects of the pandemic were not intentional or accidental on the organization’s part, and the organization was not directly a victim of an event such as a natural disaster, rumor, product tampering, or hacking. It is also unlikely that the SCCT crisis types would be relevant for other organizations at the center of the crisis, such as the World Health Organization (WHO). Victim and accidental crisis types were the closest fit, but their descriptions in SCCT (Coombs, 2015: 264) did not fully align with the situation. This suggests that *crisis types* as defined in the literature need to be broadened, as suggested by Boin et al. (2017).

This is not simply a matter of updating or expanding the range of *crisis events* that are recognized. The attendant crisis responsibilities listed in SCCT were found to be inapplicable and even misleading. Even though the organization studied could be deduced to have very low or minimal crisis responsibility according to the closest match crisis types, in fact it carried substantial responsibility in the minds of its staff and students, as well as society given that the university is a prominent public institution. Any wrong step in management and communication during the crisis could have caused heightened fear and even panic; triggered large-scale withdrawal from courses causing additional financial losses; disrupted socially important scientific research; and even cost lives, which would have severely damaged the reputation of the organization and lost the trust of staff and students.

A university faculty is not alone in this larger concept of crisis responsibility. For example, fire services and local government authorities were not responsible for the 2017 Grenfell Tower fire in London and, prior to the event, they enjoyed a favorable reputation (i.e. no negative intensifiers). But they were the subject of major criticism for their failure to evacuate the building once it was clear that the fire was out of control and for a lack of communication with residents during the crisis. An independent report castigated the fire services (Moore-Bick, 2019). This suggests a need for a broader understanding of crisis responsibility, which in turn necessitates a rethink of crisis response strategies. Being “to blame” for a crisis is not necessarily the primary determinant of crisis responsibility.

### Crisis response strategies for broader crisis responsibilities

The crisis response strategies identified in SCCT—namely denial, diminish, rebuild, and bolstering—were mostly irrelevant to the organization studied and are likely to be so for large numbers of other organizations that are not implicated under SCCT’s conceptualization of crisis responsibility. Only some bolstering strategies such as thanking and praising stakeholders for their help and support (ingratiation) and reminding stakeholders that the organization was not the cause, but a victim (*victimage*), were applicable in this case.

SCCT is primarily focused on responsibility related to the cause of a crisis and thereby fault, which leads to blame and reputational damage. In this case, responsibility was interpreted much more broadly by stakeholders, as noted above. Irrespective of attribution of crisis responsibility related to the cause of this crisis (which for the university was nil), stakeholders were highly dependent on the university and the faculty making timely and wise decisions to protect their health and safety, as well as balancing mitigation strategies with sustainability of teaching and research and, ultimately, financial sustainability. Also, key stakeholders such as staff and students expected a fast flow of accurate information, as well as two-way communication to hear their concerns and answer their barrage of questions.

This contradicts SCCT’s claim that victim crises result in “very low attributions of crisis responsibility” and that accidental crises result in “minimal attributions of crisis responsibility,” with only the intentional crisis cluster leading to “strong attributions of crisis responsibility” and “severe reputational threat” (Coombs, 2007c: 168, 2015: 264). A helpful approach might be to separate crisis responsibility from fault and blame, with a much wider range of organizations having responsibilities in today’s networked and interconnected world.

Lack of intentionality, accidental crisis events, and even victimage do not provide inoculation from responsibility and potential reputational damage. For example, during this crisis, two leading scientists in the faculty were outspoken and highly critical of the university and the faculty when they considered management was not moving quickly enough to close facilities. One called faculty leaders “irresponsible” in a Zoom conference (MW, 20 March 2020, personal communication). Another said in an e-mail: “Management was too slow to act” (NS, 27 March 2020, personal communication).^6^ Employee, student, media, and public expectations of the university and its Faculty of Science were high, even though it played no part in creating or spreading the COVID-19 virus.

Many, if not all organizations, carry an expectation of responsibility during many crises. The level and type of responsibilities vary in accordance with the role and functions of the organization. This suggests that at least one additional crisis type should be recognized, which could be called *ineffectual* in terms of stakeholder and public expectations irrespective of direct responsibility.

### Blowing up the “media myth”

A third operational learning in this crisis that challenges SCCT and much public relations literature on crisis management and crisis communication (e.g. Fearn-Banks, 2007), as well as many studies of risk communication (Reynolds and Seeger, 2005), relates to the role of news media. While news media, particularly TV, radio, and online news sites, played a key role in transmitting health information to the public during the pandemic, news media were found to be untrustworthy, ineffective, and their use was deemed even counter-productive for crisis communication with the organization’s key stakeholders.

National and local news media mainly focused on the announcements of political leaders, reports of infections and death rates, “big” stories such as stranded cruise ships, and controversial issues such as statements by Donald Trump. While news conferences and briefings by health authorities were reported and some practical information such as hand washing instructions was distributed, many media reports were inaccurate and sensational, rather than providing reliable and important *instructing* and *adjusting* information as required in crisis communication (Coombs, 2015: 265). The role of media may be different in the case of regional and specialist media. But even these suffer from the following limitation.

A second reason that news media are of declining importance is that their audience reach has reduced substantially, particularly among young people (Galan et al., 2019). Furthermore, trust in media has plummeted to unprecedented levels (Edelman, 2020). In short, news media do not have the reach or credibility required to communicate with many key stakeholders during a crisis. The centrality of media, particularly traditional media, to SCCT and in public relations theory generally is increasingly a dated and narrow approach and warrants review.

Social media are widely used across large sectors of society today, but these platforms also were identified as having limited effectiveness in crisis communication. Social media analysis showed that social platforms were rife with misinformation and disinformation including conspiracy stories and fake cures during the COVID-19 crisis (MacAweeney, 2020). The World Health Organization (2020b) declared COVID-19 an “infodemic” as well as a pandemic, and the United Nations referred to it as a “disinfodemic” (Posetti and Bontcheva, 2020), largely due to social media content. While social media facilitated social connection between staff and students during extended periods of working and studying from home, which arguably contributed somewhat to personal wellbeing, social media were found to be unreliable channels for communication by the organization.

In addition to the university’s online learning management system (Canvas) to which all course materials were transferred in an unprecedented effort, the most used and effective communication channels for the faculty and the university during the crisis were:

(i) *Video conferencing* applications including Zoom and Microsoft Teams, which were used by faculty leaders to communicate directly with staff during physical distancing and remote working from home. This included special “Zoom Ins” in which staff members could ask questions of senior leaders and managers in open forums;
(ii) *E-mail listservs* for targeted messages to academic, technical, and administrative staff; international students; and domestic students;
(iii) *Intranet* pages containing extensive information about remote teaching and remote working, as well as health and wellbeing. Along with video conferencing, the university’s and faculty’s intranet was a prime communication channel, with weekly and often daily updates posted to crisis-related pages during the period;
(iv) *Web site* home page messages and special pages containing information such as changed student enrollment procedures and dates;
(v) *Dean’s e-newsletter* that was written personally by the Dean and Deputy Dean;
(vi) *Telephone* for direct personal communication with major partners, funders, and also employees and students who reported symptoms or became infected with COVID-19.

During the peak of the crisis, the organization avoided news media and social media for the reasons identified. No media releases were issued and no media news conferences were held. *Owned media* in the context of the PESO model (paid, earned, shared, owned) provided reliable channels in which accuracy, relevance, and timeliness could be guaranteed. Much of the use of these channels occurred via mobile applications and devices. This suggests that crisis management and crisis communication literature that continues to place a heavy emphasis of traditional news media needs updating from a technological and strategic perspective. As Kent (2010) urged, “crisis research needs to move beyond the myopic focus on external communication to the media” (p. 708). This study affirmed Taylor and Kent’s (2007) *taxonomy of mediated crisis response* in which they identified the central role of organizational websites in crisis communication, but this too can be extended to include more focus on intranets, extranets, and direct digital communication.

### Internal and interpersonal

The preceding reflections and the summary of key management initiatives in Table 2 reinforce two other much-discussed principles—the importance of internal *organizational communication* and *interpersonal* communication. While many actions necessarily focussed on external stakeholders including customers (students), research funders, and government, internal communication was at the top of the list for management and communication, particularly in the early stages of the crisis. Without its workforce, an organization cannot deliver its services and products. In this case, mobilization of a superhuman effort was required to transition a large, mostly traditionally taught curriculum to online learning. Furthermore, teaching staff are a primary communication channels to students. Therefore, employees were the first priority in communication, and equal first with students in risk mitigation measures.

While correspondence via e-mail, listservs, and the intranet was inevitably necessary for scalable communication, interpersonal communication was expected by staff and trumped textual communication in most situations. This was particularly so following stressful events, such as the suicide of a staff member; implementation of staff reductions in late 2020; and announcement of further staff redundancies to occur in 2021. In addition to staff expectations of face-to-face engagement with senior executives despite physical distancing requirements, website URL access data gained from Google Analytics showed that real-time face-to-face video technologies gained much higher levels of staff and student engagement than e-newsletters and intranet pages—often by a factor of 10:1.

However, as the crisis wore on into the second half of 2020, screen fatigue—or what some called “Onlinitis”—became evident through staff feedback and student surveys. Long hours sitting and looking at screens in Zoom and Microsoft Teams meetings and in classes were reportedly causing back and eye strain, particularly for those working on small notebook computer screens. A student survey conducted in May–June 2020 found that a majority of students wanted to return to face-to-face classes as soon as possible (University of Technology Sydney, 2020a).

### CMT structure—public relations and the “dominant coalition”

The relatively low importance of news media, and stakeholder feedback revealing expectations that leaders and senior managers should speak directly, frankly, and authentically to stakeholders, informed the structure of the crisis management team (CMT). In contrast with Coombs’s (2014b) claim that “public relations plays a critical role in the crisis response by helping to develop the messages that are sent to various publics” ( para. 31), key messages and statements were directly crafted by senior university and faculty executives, and it was deemed essential that they spoke in their own voice. For internal communication, advice was taken from HR specialists in relation to matters such as sick and carer leave provisions, and because of the high priority given to the physical and mental wellbeing of staff and students. For example, free confidential counselling services were offered in all communications. Health and safety managers were also involved in all discussions, and IT technicians were essential to the CMT given the reliance on owned IT infrastructure. External communication with other stakeholders was also undertaken directly by senior executives via e-mail, telephone, and videoconferencing.

The faculty and university public relations professionals are responsible for writing and distributing media releases and web content in relation to staff and student achievements such as awards and grants, and they support marketing in student recruitment. However, during this extended crisis, the public relations staff of the university were sidelined because of the “paradox of the positive,” which Johnston et al. (2020: 1) identify in relation to emergency management communication. This refers to the need for organizations and their communication staff to over-emphasize their preparedness and capacity to deal with emergencies and crises in order to create stakeholder and/or public confidence and avoid panic, which results in a predominance of positive and promotional information. Also, the widely reported focus of public relations on persuasion and promotion (e.g. Moloney, 2006; Watson and Noble, 2007: 14) was evident in drafts of statements and staff presentations that were rejected in favor of frank direct address by faculty executives. While professionally produced, public relations materials were mostly found to be inappropriate in the crisis because of their overly positive and promotional tone.

The university’s and faculty’s senior executives resolved to speak “from the heart” to all stakeholders with no “gilding of the lily.” Also, the rehearsed and “air-brushed” image of spokespersons typically curated by media trainers was perceived as inauthentic. The most positive staff engagement, evidenced in the number of log-ins to Zoom briefings, online comments, and informal feedback to unit managers and staff supervisors, resulted from university and faculty leaders appearing online wearing a T-shirt, often from their home, speaking unscripted. Instead of a backdrop of executive office walls and journal-filled bookshelves, they often spoke from their lounge rooms, sometimes with children or pets walking through the background.

The reported shift of public relations toward integration with marketing (USC Annenberg, 2018) is likely to exacerbate a loss of authenticity. While practices vary around the world, this analysis argues that public relations associated with corporate and organizational communication needs to clearly distinguish its practice from marketing and promotion, and re-orientate toward authentic open communication with stakeholders in order to play a substantial role in crisis management and communication.

Experience during this crisis also confirmed the finding by Heide et al. (2018: 464) that “an organization’s communication function and its activities only represent a very small proportion of the communication carried out in and by that organization.” This suggests that studies of crisis communication need to look well beyond the public relations or corporate communication team.

### Re-conceptualizing the future, rather than re-establishing the past

A further observation and experience relates to leadership versus management. While managers focussed on stabilizing the organization and re-establishing “normal” operations, the organization’s senior leaders’ group (the most senior executives) established a project in mid-2020 to rethink the future and revise the organization’s Strategic Plan. The brief was to explore new ways of working, including increased online learning on a permanent basis balanced against preferences for face-to-face contact, and to develop new products to meet needs and opportunities created by the pandemic. From this perspective, the crisis was a catalyst for change; disruption was to be embraced rather than mitigated. Communication of this initiative and its messages to stakeholders posed challenges, as it embroiled the FET in the “paradox of the positive”—talking positively and even evangelically when people were losing their jobs and livelihood. Even when the need for and inevitability of change was accepted, the term “the new normal” became common in operational plans and discussions, reflecting a desire for an end to instability. The tension between normalization and innovation in the face of disruptions and crisis conditions is a subject to be explored in future research.

### Other lessons from “lived experience”

A final deeply personal reflection is relevant in honestly and frankly reporting the “lived experience” of the crisis. Over the course of 2020, I noted an increase in my level of alcohol consumption, evidenced first in household spending records and then in weight gain and a decline in physical fitness. Physical fitness was exacerbated by reduced exercise that resulted from working days that started early and frequently extended into the evening. Meetings and international conferences that were traditionally attended physically became online engagements, sometimes at 1 or 2 am because of time zone differences. Also, discussion of legal issues and risks related to contract terminations resulted in e-mails at all hours and on weekends, interrupting home life.

To explore whether health effects were common among the faculty’s senior executives, the author raised the matter in a FET meeting (a closed-door confidential forum). This revealed that a number of the senior executives were dealing with both physical and mental health concerns linked to stress and long working hours. Almost all agreed that the stress of being positive in the face of highly negative experiences, in order to maintain morale and motivation, was a challenge. Several reported sleeplessness caused by having to announce and implement staff redundancies. One stated: “Telling staff that their jobs are going is not a nice feeling. These are good people” (Anon, 2 November 2020, personal communication). Also, anti-management sentiment frequently expressed by staff took a toll, but was only reluctantly acknowledged. “Suck it up” and “if you can’t take the heat, stay out of the kitchen” were mantras expressed. One executive acknowledged that “you mask the pain” (Anon, 2 November 2020, personal communication).

Mental as well as physical health has rightfully received increased attention in workplaces, and the welfare of staff and students was a major focus in this instance. But this experience revealed that senior executives were not taking care of themselves, and the risk of “burn-out” and mental ill-health at the top of organizations are hidden risks in crises. This is arguably an area for further research and interventions as part of crisis management and communication.

## Conclusions

This study supports suggestions to expand crisis communication theory beyond the “managerial bias” noted by Waymer and Heath (2007) and the narrow focus on apologia and reputation protection discussed by Arendt et al. (2017). Specifically, it suggests that an expansion of SCCT is needed to include at least one additional crisis type that recognizes failure of an organization to meet stakeholder and/or societal expectations even when it is not at fault through intention, or by accident, or involved as a victim. This additional crisis type could be called “ineffectual” and applied to organizations that are a “downstream participant.” In today’s connected globalized world with increasing demands for corporate social responsibility (CSR) and commitment to *social purpose* by organizations including corporations (Business Roundtable, 2019), all organizations need to respond to crises beyond their control and their borders. Being at fault, and the presence of intensifiers such as a history of poor performance, does exacerbate reputation damage and trust erosion, and requires strategies such as apologia and remedial action. But “innocent” and blameless organizations cannot be bystanders during a crisis, or enjoy diminished expectations because they are victims. All organizations attract expectations from their stakeholders and need to live up to stated commitments. For example, employees of all organizations, whether private or public, expect their employers to keep them safe at work. *Emergent* strategic communication (Heath and Johansen, 2018) claims to serve the interests of stakeholders as well as the organization and, as noted previously, many organizations including private and public companies are looking beyond growth and profits to have a social purpose (Rodríguez-Vilá and Bharadwaj, 2017).

In so arguing, this analysis supports a proactive as well as an expanded approach, such as that applied in risk communication and risk management, rather than the mainly reactive approach associated with crisis communication that mainly responds to events an organization caused, or suffered from, with a primary focus on protecting its reputation. While recognizing difference between risk (potential injury or damage) and crisis (a manifested risk), the proactive and macro-societal approach of risk communication and risk management offers much to expand the contemporary relevance of crisis communication theory and practice, particularly pre-crisis communication that can contribute to preparedness.

While a number of studies reveal a focus on the use of news media and social media as primary communication channels in a crisis (Coombs, 2015, 2018; Fearn-Banks, 2007; Verhoeven et al., 2014), this analysis suggests that the literature needs to be updated to take account of the increasing range of technologies available to create and support owned media. At the same time, the substantial decline in the reach and influence of news media and the attempts of many to retain audiences through clickbait, sensationalism, and opinion appealing to populism (Macnamara, 2020), give further weight to the case for owned media in which the fidelity of information and messages can be assured. However, screen-based digital communication needs to take account of screen fatigue and be balanced with the human need for physical interaction.

Public relations can potentially play a significant role in crisis management and communication, but only if the practice facilitates authentic communication and is technologically up to date and proficient. Findings of the global *Communication Monitor* studies that “coping with the digital evolution” and “dealing with the speed and volume of information” are among the leading challenges faced by PR and corporate communication practitioners (Meng et al., 2019), along with reports that only 50% have high levels of competence with communication technologies (Zerfass et al., 2020: 81), are of concern in this regard. Also, the long-standing debate about whether PR should align with marketing has implications.

Benefits for the diverse field of practice are likely to accrue from interdisciplinary and even transdisciplinary integration of public relations theory on crisis communication, risk communication, and the hybrid field of crisis and emergency risk communication (CERC). A broader focus beyond the crisis types and events identified in SCCT, and beyond protection of an organization, is imperative to align with the principles of corporate social responsibility (CSR), social license to operate, and social purpose. Risk communication, and potential emergency communication, practices could benefit from relational, dialogic, and sociocultural concepts of public relations to engage interactively and collaboratively with stakeholders, rather than traditional broadcasting of risk and emergency information and messages. Such integration would open up the field and offer new avenues for research.

Beyond the numerous studies of leadership style, the leadership-management nexus during crises offers further interesting directions for study, including the balancing of normalization versus “futurization.”

Finally, the use of ethnography and autoethnography, particularly from an *emic* (inside) perspective, is advocated for the deep insights drawn from first-hand lived experience that these methodologies provide, along with exposure of the affective (emotional) dimensions undetected by rational cognitive analysis.

## Limitations

This analysis is based on examination of crisis communication in one organization of a particular type, which could be argued to be atypical. *While the reported ethnography and autoethnography are supported by records such as* minutes of meetings, archived e-mails, website and intranet statistics, *and staff feedback, corroborating independent external evidence was not available due to the confidentiality of discussions with government authorities and the lack of time to engage external advisers or evaluation during the crisis. Also, this was not seen as necessary given the expertise available in the university. However, the efficacy of the approach taken is supported by post-hoc data such as end-of-year student satisfaction levels equal to or higher than previous years* (University of Technology Sydney, 2020b); *maintenance of domestic enrollments for 2021; and a lack of any public or media criticism of the university in relation to its management or communication during the pandemic.*

## Notes

1. Coronaviruses are a family of viruses that exist in animals, some of which can transmit to human. They cause illness and disease ranging from the common cold to the potentially fatal Severe Acute Respiratory Syndrome (SARs) and the Middle East Respiratory Syndrome (MERs).

2. Natural disasters are defined as “catastrophic events with atmospheric, geological, and hydrological origins” (Xu et al., 2016).

3. The faculty executive team (FET) comprises the Dean, Deputy Dean, Associate Deans responsible for research, teaching, international, and engagement, the Faculty General Manager, and Finance Manager. Notes are taken by individuals, but minutes are not recorded, as the meetings are confidential.

4. Website and intranet statistics collected included logins, views of pages and videos, duration, and other access and usage data.

5. The term “social distancing” is widely used by clinical and medical authorities to denote physical separation between people. However, the term is criticized by many sociologists and some organizations, including the World Health Organization (Van Kerkhove, 2020). They point out that *physical* distancing, while maintaining social connection, is required.

6. Staff members are de-identified because consent to use their name was not obtained. However, the comments were not private because they were made on the organization’s communications platforms.

## References

[bibr1-2046147X21999972] Adams T Ellis C Jones S (2017) The International Encyclopedia of Communication Research Methods. New York, NY: John Wiley & Sons.

[bibr2-2046147X21999972] Arendt C LaFleche M Limperopulos M (2017) A qualitative meta-analysis of apologia, image repair, and crisis communication: Implications for theory and practice. Public Relations Review 43(3), 517–526.

[bibr3-2046147X21999972] Benoit W (1995) Accounts, Excuses, and Apologies: A Theory of Image Restoration. New York, NY: University of New York Press.

[bibr4-2046147X21999972] Boin A Hart P Stern E , et al. (2017) The Politics of Crisis Management: Public Leadership Under Pressure. Cambridge: Cambridge University Press.

[bibr5-2046147X21999972] Bullough R Pinnegar S (2001) Guidelines for quality autobiographical forms of self-study research. Educational Researcher 30(2): 3–21.

[bibr6-2046147X21999972] Bundy J Pfarrer M Short C , et al. (2017) Crises and crisis management: Integration, interpretation, and research development. Journal of Management 43(6): 1661–1692.

[bibr7-2046147X21999972] Business Roundtable (2019) Statement on the purpose of a corporation. Available at: https://opportunity.businessroundtable.org/wp-content/uploads/2019/08/Business-Roundtable-Statement-on-the-Purpose-of-a-Corporation-with-Signatures.pdf (accessed 10 November 2020).

[bibr8-2046147X21999972] Cameron G Pang A Yin Y (2008) Contingency theory. In: Hansen-Horn T Neff B (eds) Public Relations from Theory to Practice. Boston, MA: Pearson, pp.134–157.

[bibr9-2046147X21999972] Coombs WT (2006) Crisis management: A communicative approach. In: Botan C Hazelton V (eds) Public Relations Theory II. Mahwah, NJ: Lawrence Erlbaum, pp.149–173.

[bibr10-2046147X21999972] Coombs WT (2007a) Ongoing Crisis Communication: Planning, Managing, and Responding, 2nd edn. Thousand Oaks, CA: Sage.

[bibr11-2046147X21999972] Coombs WT (2007b) Crisis management and communications. Institute for Public Relations. Available at: https://instituteforpr.org/crisis-management-and-communications (accessed 10 November 2020).

[bibr12-2046147X21999972] Coombs WT (2007c) Protecting organization reputations during a crisis: The development and application of situational crisis communication theory. Corporate Reputation Review 10: 163–176.

[bibr13-2046147X21999972] Coombs WT (2014a) Applied Crisis Communication and Crisis Management. Thousand Oaks, CA: Sage.

[bibr14-2046147X21999972] Coombs WT (2014b) Crisis management and communications. Institute for Public Relations. Available at: https://instituteforpr.org/crisis-management-communications (accessed 10 November 2020).

[bibr15-2046147X21999972] Coombs WT (2015). Situational theory of crisis: Situational crisis communication theory and corporate reputation. In: Carroll C (ed) The Handbook of Communication and Corporate Reputation. Malden, MA: Wiley Blackwell, pp.262–278.

[bibr16-2046147X21999972] Coombs WT (2018) Revising situational crisis communication theory: The influences of social media on crisis communication theory and practice. In: Austin L Jin Y (eds) Social Media and Crisis Communication. New York, NY: Routledge, pp.21–39.

[bibr17-2046147X21999972] Coombs WT (2019) Ongoing Crisis Communication: Planning, Managing, and Responding, 5th edn. Thousand Oaks, CA: Sage.

[bibr18-2046147X21999972] Covello V (1992) Risk communication: An emerging area of health communication research. In: Deetz S (ed) Communication Yearbook, vol. 15. New York, NY: Routledge, pp.359–373.

[bibr19-2046147X21999972] Creswell J (2009) Research Design: Qualitative, Quantitative and Mixed Methods Approaches, 3rd edn. Thousand Oaks, CA: Sage.

[bibr20-2046147X21999972] Deephouse D (2000) Media reputation as a strategic resource: An integration of mass communication and resource-based theories. Journal of Management 26: 1091–1112.

[bibr21-2046147X21999972] Denzin N Lincoln Y (eds) (2008) Strategies of Qualitative Inquiry. Thousand Oaks, CA: Sage.

[bibr22-2046147X21999972] Department of Health (2020) Coronavirus (COVID-19) at a glance. Available at: https://www.health.gov.au/resources/publications/coronavirus-covid-19-at-a-glance-31-december-2020 (accessed 31 December 2020).

[bibr23-2046147X21999972] Edelman (2020) 2020 Edelman Trust Barometer. Available at https://www.edelman.com/trustbarometer (accessed 10 November 2020).

[bibr24-2046147X21999972] Eriksson J (ed) (2017) Threat Politics: New Perspectives on Security, Risk and Crisis Management. Abingdon: Routledge.

[bibr25-2046147X21999972] Fall L (2004) The increasing role of public relations as a crisis management function: An empirical examination of communication re-strategizing efforts among destination organization managers in the wake of 11th September, 2001. Journal of Vacation Marketing 10(3): 238–252.

[bibr26-2046147X21999972] Fearn-Banks K (2007) Crisis Communications: A Casebook Approach, 3rd edn. Mahwah, NJ: Lawrence Erlbaum.

[bibr27-2046147X21999972] Fransden F Johansen W (2010) Apologizing in a globalizing world: Crisis communication and apologetic ethics. Corporate Communications: An International Journal 15(4): 350–364.

[bibr28-2046147X21999972] Galan L Osserman J Parker T , et al. (2019) How Young People Consume News and the Implications for Mainstream Media. Oxford: Reuters Institute for the Study of Journalism, Oxford University. Available at: https://reutersinstitute.politics.ox.ac.uk/our-research/how-young-people-consume-news-and-implications-mainstream-media (accessed 10 November 2020).

[bibr29-2046147X21999972] Geertz C (1973) Thick description: Toward an interpretive theory of culture. In: Geertz C (ed) The Interpretation of Cultures: Selected Essays. New York, NY: Basic Books, pp.3–30.

[bibr30-2046147X21999972] Greenhouse C (2002) Introduction: Altered states, altered lives. In: Greenhouse C Mertz E Warren K (eds) Ethnography in Unstable Places: Everyday Lives in Contexts of Dramatic Political Change. Durham, NC: Duke University Press, pp.1–34.

[bibr31-2046147X21999972] Haddow G Bullock J Coppola D (2017) Emergency Management, 6th edn. Amsterdam, Netherlands: Elsevier.

[bibr32-2046147X21999972] Heath R (2010) Crisis communication: Defining the beast and de-marginalizing key publics. In: Coombs W Holladay S (eds) The Handbook of Crisis Communication. Malden, MA: Wiley-Blackwell, pp.1–14.

[bibr33-2046147X21999972] Heath R Johansen W (eds) (2018) The International Encyclopedia of Strategic Communication. New York, NY: Wiley-Blackwell

[bibr34-2046147X21999972] Heide M von Platen S Simonsson C , et al. (2018) Expanding the scope of strategic communication: Towards a holistic understanding of organizational complexity. International Journal of Strategic Communication 12(4): 452–468.

[bibr35-2046147X21999972] Heider F (1958) The Psychology of Interpersonal Relations. New York, NY: Wiley.

[bibr36-2046147X21999972] International Labour Organization (2020) COVID-19 leads to massive labour income losses worldwide. Available at: https://www.ilo.org/global/about-the-ilo/newsroom/news/WCMS_755875/lang–en/index.htm (accessed 11 November 2020).

[bibr37-2046147X21999972] Johnston K Taylor M Ryan B (2020) Emergency management communication: The paradox of the positive in public communication preparedness. Public Relations Review 46(2): 101903.

[bibr38-2046147X21999972] Kent M (2010) What is a public relations “crisis”? Refocusing crisis research. In: Coombs W Holladay S (eds) The Handbook of Crisis Communication. Malden, MA: Wiley-Blackwell, pp.705–712.

[bibr39-2046147X21999972] L’Etang J Hodges C Pieczka M (2012) Cultures and places: Ethnography in public relations spaces. Public Relations Review 38(4): 519–521.

[bibr40-2046147X21999972] Laufer D Wang Y (2018) Guilty by association: The risk of crisis contagion. Business Horizons 61: 173–179.

[bibr41-2046147X21999972] Lerbinger O (1997) The Crisis Manager: Facing Risk and Responsibility. Mahwah, NJ: Lawrence Erlbaum.

[bibr42-2046147X21999972] Lerbinger O (2012) The Crisis Manager: Facing Disaster, Conflicts and Failures. New York, NY: Routledge.

[bibr43-2046147X21999972] Lunenberg F (2011) Leadership versus management: A key distinction—At least in theory. International Journal of Management, Business, and Administration 14(1): 1–4.

[bibr44-2046147X21999972] MacAweeney E (2020) Who benefits from health misinformation? Medium, Data & Society Institute. Available at: https://points.datasociety.net/who-benefits-from-health-misinformation-8d094804058d (accessed 30 March 2020).

[bibr45-2046147X21999972] Macnamara J (2020) Beyond Post-communication: Challenging Disinformation, Deception, and Manipulation. New York, NY: Peter Lang.

[bibr46-2046147X21999972] Maréchal G (2010) Autoethnography. In: Mills A Durepos G Wiebe E (eds) Encyclopedia of Case Study Research, vol. 2. Thousand Oaks, CA: Sage, pp.43–45.

[bibr47-2046147X21999972] Meng J Reber B Berger B , et al. (2019) North American Communication Monitor 2018-2019. Tracking Trends in Fake News, Issues Management, Leadership Performance, Work Stress, Social Media Skills, Job Satisfaction and Work Environment. Tuscaloosa, AL: The Plank Center for Leadership in Public Relations.

[bibr48-2046147X21999972] Moloney K (2006) Rethinking Public Relations: PR, Propaganda, and Democracy. Abingdon: Routledge.

[bibr49-2046147X21999972] Moore-Bick M (2019) Grenfell tower inquiry report. Available at: https://www.grenfelltowerinquiry.org.uk/phase-1-report (accessed 10 November 2020).

[bibr50-2046147X21999972] Moreno A Navarro C Alkazemi M (2018) How the public and public relations professionals interpret leadership in Spain: Results from the ComGap study. Corporate Communications: An International Journal 23(1): 84–99.

[bibr51-2046147X21999972] Neuman W (2006) Social Research Methods: Qualitative and Quantitative Approaches, 6th edn. Boston, MA: Pearson.

[bibr52-2046147X21999972] Posetti J Bontcheva K (2020) Disinfodemic: Deciphering COVID-19 Disinformation. Policy Brief 1. UNESCO. Available at: https://en.unesco.org/covid19/disinfodemic (accessed 10 November 2020).

[bibr53-2046147X21999972] Quacquarelli Symonds (2020) World university rankings. Available at: https://www.topuniversities.com/university-rankings/world-university-rankings/2020 (accessed 10 November 2020).

[bibr54-2046147X21999972] Renn O (2008) Risk communication: Insights and requirements for designing successful communication programs on health and environmental hazards. In: Heath R O’Hair H (eds) Handbook of Risk and Crisis Communication. London: Taylor and Francis, pp.80–98.

[bibr55-2046147X21999972] Reynolds B Seeger M (2005) Crisis and emergency risk communication as an integrative model. Journal of Health Communication 10(43): 43–55. 15764443 10.1080/10810730590904571

[bibr56-2046147X21999972] Rodríguez-Vilá O Bharadwaj S (2017) Competing on social purpose: Brands that win by tying mission to growth. Harvard Business Review, September-October. Available at: https://hbr.org/2017/09/competing-on-social-purpose (accessed 4 January 2020).

[bibr57-2046147X21999972] Schwarz A (2008) Covariation-based causal attributions during organizational crises: Suggestions for extending situational crisis communication theory (SCCT). International Journal of Strategic Communication 2(1): 31–53.

[bibr58-2046147X21999972] Taylor M Kent M (2007) Taxonomy of mediated crisis response. Public Relations Review 33(1): 140–146.

[bibr59-2046147X21999972] Tedlock B (2000) Ethnography and ethnographic representation. In: Denzin N Lincoln Y (eds) Handbook of Qualitative Research. Thousand Oaks, CA: Sage, pp.455–486.

[bibr60-2046147X21999972] Tedlock B (2008) The observation of participation and the emergence of public ethnography. In: Denzin N Lincoln Y (eds) Strategies of Qualitative Inquiry, 3rd edn. Thousand Oaks, CA: Sage, pp.151–171.

[bibr61-2046147X21999972] University of Technology Sydney (2020a) Student survey report to the UTS Sustainability Committee.

[bibr62-2046147X21999972] University of Technology Sydney (2020b) Student feedback survey 2020.

[bibr63-2046147X21999972] USC Annenberg (2018) 2018 global communications report. Available at: https://annenberg.usc.edu/research/center-public-relations/global-communications-report (accessed 10 November 2020).

[bibr64-2046147X21999972] Van Kerkhove M (2020) Transcript of News Media Conference with Discussion of Physical Distancing Versus Social Distancing. Geneva: World Health Organization. Available at: https://www.who.int/docs/default-source/coronaviruse/transcripts/who-audio-emergencies-coronavirus-press-conference-full-20mar2020.pdf?sfvrsn=1eafbff_0 (accessed 10 November 2020)

[bibr65-2046147X21999972] Verhoeven P Tench R Zerfass A , et al. (2014) Crisis? What crisis? How European professionals hand crises and crisis communication. Public Relations Review 40(1): 107–109.

[bibr66-2046147X21999972] Wall S (2008) Easier said than done: Writing an autoethnography. International Journal of Qualitative Methods 7(1): 38–53.

[bibr67-2046147X21999972] Watson T Noble P (2007) Evaluating Public Relations: A Best Practice Guide to Public Relations Planning, Research and Evaluation, 2nd edn. London: Kogan Page.

[bibr68-2046147X21999972] Waymer D Heath R (2007) Emergent agents: The forgotten publics in crisis communication and issue management research. Journal of Applied Communication Research 35(1): 88–108.

[bibr69-2046147X21999972] Waymer D Logan N (2016) Extending organizational memory and corporate communications research via autoethnography/autobiography. The Qualitative Report 21(8):1457–1474.

[bibr70-2046147X21999972] World Health Organization (2020a) WHO director-general’s opening remarks at the media briefing on COVID-19. Available at: https://www.who.int/dg/speeches/detail/who-director-general-s-opening-remarks-at-the-media-briefing-on-covid-19—11-march-2020 (accessed 10 November 2020).

[bibr71-2046147X21999972] World Health Organization (2020b) Novel coronavirus (2019-nCoV). Situation report 13. Available at: https://www.who.int/docs/default-source/coronaviruse/situation-reports/20200202-sitrep-13-ncov-v3.pdf (accessed 10 November 2020).

[bibr72-2046147X21999972] Worldometer (2020) Coronavirus cases. Available at: https://www.worldometers.info/coronavirus (accessed 10 November 2020).

[bibr73-2046147X21999972] Xu J Wang Z Shen F , et al. (2016) Natural disasters and social conflict: A systematic literature review. International Journal of Disaster Risk Reduction 17: 38–48.

[bibr74-2046147X21999972] Zerfass Verhoeven P Moreno A , et al. (2020) European Communication Monitor 2020. Ethical Challenges, Gender Issues, Cyber Security, and Competence Gaps in Strategic Communication. Results of a Survey in 44 Countries. Brussels: EUPRERA/EACD.

